# The decentralized academic health center: a 21st-century model for rural health innovation

**DOI:** 10.1093/haschl/qxag107

**Published:** 2026-05-09

**Authors:** Patrick C Hardigan, Johannes Vieweg

**Affiliations:** College of Health Sciences, University of Wyoming, Laramie, WY 82071, USA; Mass General Brigham, Harvard University, Cambridge, MA, USA

**Keywords:** Decentralized Academic Health Center, D-AHC, rural health care, rural workforce development, academic health centers, digital health, shared governance, interoperable data systems, community engagement, rural health transformation

## Abstract

Rural communities in the United States face persistent gaps in health care access, workforce capacity, and outcomes despite substantial federal investment. Academic health centers (AHCs) are central to clinical care, research, and education, but remain largely urban-based and structurally misaligned with rural needs. We propose the Decentralized Academic Health Center (D-AHC), a distributed model that integrates academic institutions, rural providers, public health systems, and communities through shared governance, interoperable data infrastructure, and digital health tools. Unlike traditional hub-and-spoke systems, the D-AHC treats rural partners as co-equal participants in education, research, and care delivery. Using Wyoming as a case example, we outline 5 implementation domains: workforce development, research and data systems, clinical partnerships and digital health, community engagement and policy, and financial sustainability. We further describe provider and community readiness considerations and present a practical implementation roadmap. The D-AHC aligns with major federal initiatives, including HRSA rural investments and the 2025 Rural Health Transformation Program, offering a scalable framework for improving access, strengthening workforce pipelines, and enhancing system sustainability in rural and frontier regions.

Key points What is already known?

- AHCs are central to innovation but remain urban-focused.

What does this study add?

- Introduces a decentralized, network-based academic model.

What are the policy implications?

- Supports implementation of federal rural health investments through structural redesign.

## Introduction

Academic health centers (AHCs) are foundational to the US health system, serving as hubs for medical education, specialty care, and biomedical research.^1^ However, most AHCs are concentrated in urban areas and organized around centralized governance and infrastructure.^2^ Estimates suggest that the vast majority of AHC clinical and research capacity is located in metropolitan regions, despite 15%-20% of the US population residing in rural areas.^3^

This structural mismatch contributes to persistent rural health disparities. Rural populations experience higher rates of chronic disease, maternal morbidity, and premature mortality, alongside limited access to primary care, behavioral health, and specialty services.^4-6^ Workforce shortages and hospital closures further exacerbate these inequities.^5^

Federal investment in rural health has expanded significantly. The Health Resources and Services Administration (HRSA) has increased funding for workforce, telehealth, and rural system capacity.^7^ The National Institutes of Health's AIM-AHEAD initiative supports artificial intelligence (AI) and data infrastructure in underserved communities.^8^

Most notably, the 2025 Rural Health Transformation Program (RHTP) authorizes up to $50 billion over 5 years to support state-led rural health innovation.^9^ However, without structural redesign, these investments risk reinforcing centralized delivery models that inadequately serve rural communities.

We propose the Decentralized Academic Health Center (D-AHC), a distributed model that integrates rural providers, public health systems, and communities as co-equal partners. This approach aligns institutional structure with the geographic and social realities of rural health.

## Conceptual model

**Figure 1.**
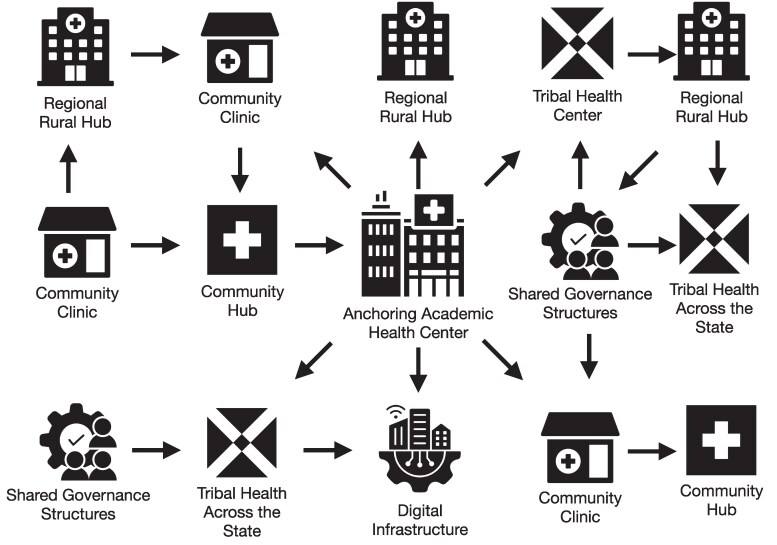
Conceptual model of a decentralized academic health center. A distributed network linking academic institutions, rural hubs, community organizations, and digital infrastructure through shared governance and bidirectional data exchange.

The D-AHC reconceptualizes the AHC as a distributed, multinodal network rather than a single institutional hub—Figure 1.

Core components include:

- Academic anchor institutions
- Regional rural hubs (eg, critical access hospitals)
- Community-based organizations
- Digital infrastructure (data platforms, telehealth systems)

These nodes are connected through:

- Shared governance
- Interoperable data systems
- Bidirectional information exchange

This structure contrasts with traditional hub-and-spoke models, which centralize authority and limit rural participation.^10^ The D-AHC instead functions as a learning health system, where all nodes contribute to care delivery, education, and research.

## Methods

This analysis is based on a structured review of literature and policy documents (2006-2025) identified through PubMed, Google Scholar, and federal agency sources (eg, HRSA, NIH, AAMC). Approximately 50 sources were selected based on relevance to rural health systems, academic medicine, and digital health.

A structured review was chosen over a formal systematic review because the objective was conceptual synthesis rather than exhaustive evidence aggregation. This approach allows integration of policy reports, emerging frameworks, and multidisciplinary literature not typically captured in systematic reviews.

Limitations include potential selection bias and a lack of formal quality appraisal. However, these are consistent with policy-oriented conceptual work and are mitigated through reliance on widely cited and authoritative sources.

## Comparative framework

Traditional AHCs are characterized by centralized governance, urban concentration, and referral-based relationships with rural providers. In contrast, the D-AHC distributes authority, integrates data systems, and embeds academic functions in rural settings—See Table 1.

**Table 1.** Comparison of traditional AHC and D-AHC.

Dimension	Traditional centralized academic health center	De-centralized academic health center model
**Structure**	Single urban campus with peripheral rural affiliates; linear hub-spoke referrals	Multinodal network: anchoring academic hubs, regional rural nodes (eg, tribal centers), and digital infrastructure; bidirectional/lateral connections
**Governance**	Centralized decision-making; rural sites advisory only	Shared federated councils with rural co-chairs and local veto on priorities; aligns with AAMC integration goals via equitable representation
**Data/integration mechanism**	Siloed urban data systems; one-way rural data uploads	Shared data lake with AI analytics for real-time equity monitoring; enables co-production (eg, rural-led trials), differing from AAMC outreach by flattening hierarchies
**Education/workforce**	Urban-based curricula with short rural rotations	Distributed longitudinal pathways (eg, tele-simulation); rural faculty as core partners, advancing AAMC rural training
**Research**	Urban-centric trials; rural recruitment episodic	Embedded pragmatic studies co-led by rural nodes; addresses AAMC equity pillars via community-governed AI
**Clinical care**	Urban specialties dominate; rural sites for basics	Mesh telehealth backbone with AI triage; reduces transfers, builds on AAMC partnerships for mutual sustainability
**Equity outcomes**	Exacerbates urban-rural gaps (eg, broadband silos)	Prioritizes digital equity (eg, co-designed tools); measurable via dashboards, fulfilling AAMC calls for rural lifeline integration

Key distinctions include:

- Governance: centralized vs shared
- Data: siloed vs interoperable
- Education: episodic rural exposure vs longitudinal rural training
- Research: urban-centric vs community-engaged
- Care delivery: referral-based vs integrated digital networks

## Implementation domains

### Teaching and workforce

The D-AHC prioritizes rural workforce development through distributed training pathways and “grow-your-own” programs.^11^

Key elements include:

- Longitudinal rural clinical training
- Interprofessional education models
- Tele-simulation and remote learning platforms
- Recognition of rural clinicians as core faculty

This approach addresses workforce shortages while improving retention in rural communities.

### Research and data

The model incorporates shared data infrastructure integrating clinical, public health, and social determinants data.^12^

AI-enabled tools support:

- Population health management
- Identification of service gaps
- Predictive modeling for resource allocation

Research shifts toward community-engaged and practice-based studies conducted in rural settings, aligning with federal priorities on equity and local data governance.^8^

### Clinical partnerships and digital health

The D-AHC includes a telehealth backbone connecting rural and academic sites through services such as:

- Tele-ICU
- Tele-behavioral health
- Virtual specialty care

Standardized care pathways and shared clinical governance improve quality while maintaining local autonomy.^10^

### Community engagement and policy

Community participation is embedded through regional governance structures that include patients, public health agencies, and local organizations.

Unlike traditional models, which often operate parallel to policy frameworks, the D-AHC is designed to actively align with evolving policy environments, including:

- Telehealth reimbursement policies
- Workforce development programs
- State-led transformation initiatives (eg, RHTP)

This alignment ensures that funding mechanisms and regulatory frameworks reinforce, rather than constrain, decentralized models.

### Financial sustainability

The D-AHC diversifies revenue through:

- Federal funding (eg, HRSA, RHTP)
- Value-based payment models
- Public–private partnerships

This reduces reliance on inpatient revenue and supports long-term rural system viability.^5^

### Provider and community readiness

Successful implementation depends on provider and community engagement.

**Provider considerations:**

- Reduced professional isolation through network participation
- Access to specialty support and continuing education
- Concerns about workload distribution and compensation

**Rural system readiness:**

- Variable data infrastructure across sites
- Limited research capacity in some settings
- Need for technical and workforce support

**Community capacity:**

- Strong local leadership in many regions
- Need for investment in workforce pipelines
- Importance of culturally grounded approaches, particularly in tribal communities

Targeted investment in infrastructure, training, and governance can address these gaps.

## Discussion

The D-AHC aligns with major national policy priorities. HRSA investments and the RHTP provide unprecedented funding for rural health transformation.^7,9^

However, these investments require structural models capable of integrating care, education, and research across distributed systems. The D-AHC provides such a framework.

Digital health and AI offer significant opportunities but also pose risks related to equity.^12^ The D-AHC mitigates these risks through shared governance and community-informed implementation.

## Policy implications and implementation roadmap

For institutions pursuing a D-AHC, implementation should proceed in phases:

### Phase 1: assessment and partnership development

- Conduct multistakeholder needs assessment
- Identify regional partners (hospitals, clinics, public health)
- Establish shared vision and governance principles

### Phase 2: governance and infrastructure

- Create regional governance councils
- Develop data-sharing agreements
- Invest in interoperable digital platforms

### Phase 3: pilot programs

- Launch targeted initiatives (eg, telehealth services, rural training tracks)
- Focus on high-need areas (behavioral health, maternal health)
- Establish metrics for evaluation

### Phase 4: scaling and integration

- Expand successful pilots across the network
- Integrate research and education functions
- Align with state and federal funding opportunities

### Phase 5: evaluation and sustainability

- Monitor outcomes (access, workforce, quality)
- Adjust governance and operations
- Transition to sustainable funding models

## Conclusion

The Decentralized Academic Health Center represents a structural redesign of academic medicine for rural and frontier regions. By integrating education, research, and clinical care across distributed networks, the model aligns academic systems with rural health needs.

Current federal investments create a timely opportunity to test and scale this model. With appropriate governance, infrastructure, and community engagement, the D-AHC can improve access, strengthen workforce capacity, and enhance long-term sustainability in rural health systems.
